# Diaries as “Soul Portraits”? Interpretation and Theorization of Adolescents’ Self-Descriptions in the German-Speaking Youth Psychology of the 1920s and 1930s

**DOI:** 10.1007/s00048-021-00308-5

**Published:** 2021-09-07

**Authors:** Carla Seemann

**Affiliations:** Chair of Romance Cultural Studies, Saarland University, 66123 Saarbrücken, Germany

**Keywords:** Youth psychology, Diary writing, Adolescent development, Puberty, Charlotte Bühler, William Stern, Siegfried Bernfeld, Jugendpsychologie, Tagebuch schreiben, Jugendliche Entwicklung, Pubertät, Charlotte Bühler, William Stern, Siegfried Bernfeld

## Abstract

In the first two decades of the twentieth century, the figure of the adolescent (*Jugendlicher*) was introduced into public discourse in the German-speaking world. The adolescent soon became an epistemic object for the still loosely defined field of psychology. Actors in the slowly differentiating scientific field of youth psychology were primarily interested in the normal development of adolescent subjects and sought out new materials and methods to research the inner life of young people. In order to access this inner life, they turned to the interpretation of diaries and other self-descriptions. This article takes up the questions of how diaries were used in the scientific context of psychology, and how diary writing was psychologically interpreted and theorized. The theoretical and methodological contexts of psychological knowledge production grouped around the subject of the diary will be examined in keeping with Hans-Jörg Rheinberger’s concept of historical epistemology. This analysis is carried out by using the example of three central actors who were in conversation with each other during the 1920s and 1930s: the developmental psychologist Charlotte Bühler (1893–1974), the psychologist and founder of personalistic psychology William Stern (1871–1938), and the youth activist Siegfried Bernfeld (1892–1953), who was influenced by Freudian psychoanalysis.

## Abstract (de)

In den ersten zwei Jahrzehnten des 20. Jahrhunderts rückte die Figur des/der Jugendlichen im deutschsprachigen Raum in den Fokus des öffentlichen Diskurses. Zunächst in anwendungsbezogenen Bereichen im Sinne einer Problematisierung vorgeformt, wurde sie bald zu einem epistemischen Objekt für das noch lose Feld der Psychologie. Die Akteur:innen jener sich langsam ausdifferenzierenden Jugendpsychologie waren vornehmlich an der Normalentwicklung jugendlicher Subjekte interessiert und suchten nach neuen Materialien und Methoden zur Erforschung jugendlicher Innerlichkeit. Prominent vertreten und schnell etabliert war die Deutung von Tagebüchern und anderen Selbstzeugnissen junger Menschen. Im Zentrum dieses Textes steht die Frage, wie Tagebücher in einem wissenschaftlichen Kontext der Psychologie verwendet wurden und wie das Tagebuchschreiben somit psychologische Interpretationen und Theoretisierungen erfuhr. Dafür werden im Sinne der Historischen Epistemologie Hans-Jörg Rheinbergers die theoretischen und methodischen Kontexte der um den Gegenstand Tagebuch gruppierten psychologischen Wissensproduktion in den Blick genommen. Dies geschieht am Beispiel dreier zentraler und miteinander in Diskussion stehender Akteur:innen: der Entwicklungspsychologin Charlotte Bühler (1893–1974), des Psychologen William Stern (1871–1938) sowie des von der Freud’schen Psychoanalyse beeinflussten Jugendaktivisten Siegfried Bernfeld (1892–1953).

*The most intimate document of adolescents’ inner life is the diary. The most secret emotions, and especially the secret emotions of the soul, are communicated to the diary like a secret friend.*^1^ (Lau 1927: 301)

In January 1911, a young girl, later known in psychological circles under the pseudonym Irmgard Winter, began her first diary: “My dear good Fräulein Wörner! Since I met you, I’ve been happy. Why wasn’t I before? Actually, it’s because I’m ungrateful. Everything is so beautiful” (Bühler 1922: 3). By the time she was 17 she would fill three more volumes. But only the first volume would ever be seen by a wide audience: The German developmental psychologist Charlotte Bühler (1893–1974) got hold of it and published it in 1922 under the title *Tagebuch eines jungen Mädchens*. In this transmission to the scientific realm, the diary took on a different meaning: it no longer was a private book filled with heartaches and worries, but it became an intimate document for research purposes that could reveal the secret inner life of young people, as the psychologist Ernst Lau (1893–1978) put it in his 1927 outline “Über Methoden und Ergebnisse der Jugendkunde.”

How did psychologists in the 1920s and 1930s use, interpret, and theorize diaries in their research? In this article, I offer a comparative analysis of the psychological research on diaries of young people in the 1920s and 1930s in the works of Charlotte Bühler, William Stern (1871–1938), and Siegfried Bernfeld (1892–1953). Three monographs using diaries as a basis for a psychology of adolescence form the material for the study: Bühler’s *Das Seelenleben des Jugendlichen *(1922), Stern’s *Anfänge der Reifezeit. Ein Knabentagebuch in psychologischer Bearbeitung *(1925a), and Bernfeld’s *Trieb und Tradition im Jugendalter. Kulturpsychologische Studien an Tagebüchern *(1931). In these monographs, as well as in several articles and reviews published in psychological journals, Bühler, Stern, and Bernfeld referred to and commented on each other’s publications and engaged in a heated debate over how to edit, publish, and interpret young people’s diaries.

In the first part, I analyze the theoretical background of Bühler’s, Stern’s, and Bernfeld’s view on adolescence. Secondly, I investigate how this theoretical background is intertwined with the methodological approach and the way these psychologists interpreted diaries as sources for the adolescent’s inner life. Thirdly, I show how theoretical background and means of interpretation were mirrored in the way they edited and published their monographs. Finally, I conclude with a broader reflection on the functions that the diary fulfilled in the field of psychology of the 1920s and 1930s and consider why its prominence in the world of psychological research was only slowly replaced by other types of material.

Ernst Lau’s survey article (1927) on methods of the still-forming field of youth psychology shows that five years after Bühler’s publication, the study of young people’s diaries had arrived in the scientific mainstream. It had become one method among several others, including statistics, questionnaires, or external observations. However, it is noteworthy that especially Bühler was able to make a name for herself with her research on diaries; the psychological use and interpretation of diaries was referred to by other researchers as the “Bühler Method” (see for example Nobiling 1929: 48).With her diary-based research, Charlotte Bühler had quickly established herself in the scientific world: After having studied psychology in Berlin and Munich, she followed her husband Karl Bühler (1879–1963) to Vienna in 1923, where the two founded the Vienna Psychological Institute. It was in this setting that she pursued the systematic acquisition of diaries (*Wiener Tagebuchsammlung*) and a large portion of her 1920s publications used diaries of this collection as a material basis. In contrast to Bühler, William Stern is better known for his works on child psychology and his theory of critical personalism (*Kritischer Personalismus*) than for his contribution to youth psychology. Through individual lectures, articles,^2^ and radio contributions he took up the subject of adolescence. Stern considered his 1925 publication *Anfänge der Reifezeit. Ein Knabentagebuch in psychologischer Bearbeitung* as preparatory work for a monograph on the psychology of maturing youth that was planned, but never published (see Heinemann 2016: 288). Like Bühler, Stern was institutionally well anchored: From 1906 on he was head of the *Institut für angewandte Psychologie und psychologische Sammelforschung* in Kleinglienicke close to Berlin (see Lipmann & Stern 1907), while at the same time he held a chair in philosophy at the University of Breslau. From 1916 to 1933 he was director of the Hamburg Psychological Institute, which, like Bühler’s Institute, was a leading institution in several research areas such as the fields of youth psychology and psychological pedagogy. Due to the racist legislation of National Socialism, Stern was forced to emigrate in 1933, first to the Netherlands and later to the US. In contrast to Stern and Bühler, Siegfried Bernfeld had never pursued an academic career and his work was thus rarely noted by the established psychologists of the period. After having studied pedagogy, psychology, philosophy and sociology, and obtaining a PhD in 1915, Bernfeld entered the psychoanalytic circles around Freud and worked as a psychoanalyst at the beginning of the 1920s. Beginning with his 1915 dissertation, he was interested in the subject of adolescence and also published texts on questions of psychoanalysis, pedagogy, and education. Furthermore, he was a political activist in the circles of the Viennese Youth Culture Movement (*Jugendkulturbewegung*). This movement aimed to intensify academic research on adolescence undertaken by young people themselves, and to spread youth culture. Bernfeld realized both aims by establishing an Archive for Youth Culture (*Archiv für Jugendkultur*) in 1913, which also became a central point of collection for diaries.

Psychologists’ interest in adolescence arose out of the great attention paid to youth in other social and non-academic fields in the first two decades of the twentieth century. In medicine, law, and curative education a discourse of problematization and deviance was constructed around the adolescent subject (see Dudek 1990: 49–83). In the psychological context of the 1920s, however, this discourse shifted decisively: Psychologists like Bühler and Stern became increasingly interested in what they thought of as “normal” developments in adolescence instead of thinking of this phase of life as a crisis. This discourse was largely decoupled from the earlier psychopathological knowledge on adolescence and normalized in a loosely defined field of “youth psychology.” In the search for methods appropriate to the new object of research, psychologists often reached for everyday practices and translated them into the canon of science, as Laurens Schlicht (2020) has shown for graphology.

The case is similar for the use of the diary in youth psychology at the beginning of the twentieth century. The reasons for psychologists’ attention to the diary cannot be explained without considering its cultural significance as an everyday practice, which had also made diaries interesting for other scientific disciplines. Historians, for example, discussed the value of autobiographies for their research in the same period, and tried to evaluate their “authenticity” (see for example Glagau 1903). As can be seen in Graf’s & Steuwer’s (2015) article, reflection on the methodological uses of diaries in history continues today. According to them, diaries still are read primarily as “authentic” sources, but hardly ever merit scholarly study on their own. Against this background, Graf & Steuwer show that the idea of authenticity itself is historically produced, and strongly bound to understandings of diary writing as social practice.

The perception of diary writing at the beginning of the twentieth century carried certain cultural and social markers acquired before it entered the academic realm. Cultural historians have shown that in comparison to the writing practices that preceded it, diary writing was characterized by a new “self-feeling.”^3^ In the eighteenth century, this practice was used in the context of pietism as a means to examine one’s own conscience, control and confess one’s own (mis-)behavior and, thus, receive absolution. At the end of each day, behavior and thoughts had to be written down honestly. The ideas of secrecy, self-discipline and self-exploration became strongly attached to diary writing in this period and remained just as potent over the course of the next century, when the practice became secularized and popularized amongst the bourgeoisie and within the sphere of literature. Gerhalter & Hämmerle (2015) pointed out that while only diaries of male authors were canonized and published in the nineteenth century, bourgeois women also engaged in diary writing while confined to the private sphere. Entrepreneurs promoted and encouraged this activity by launching pre-structured, ready-to-use diaries specifically produced for women. At the beginning of the twentieth century, diary writing spread more broadly across different classes and genders—especially among young people—when it was implemented in school curricula. Another reason why young people became attracted to diaries was their dissemination through the exploding genre of autobiographic literature—often written in diary form—after the First World War. Thus, it can be assumed that the connection between diary writing, exploring of the inner self, and adolescence had already been made before psychologists used this connection and theorized it.

The history of psychology offers some analyses of the emergence of different psychological research practices and the way research objects and techniques are constructed. A perspective which investigates and problematizes the conditions of scientific knowledge production comes from the field of historical epistemology. The work of Hans-Jörg Rheinberger (1997) offers one approach to this field. Rheinberger argued in the context of the natural sciences and their “experimental systems” that research objects were not pre-existing entities that are to be simply found or discovered during the research process. Instead, he analyzed how the instruments of research (the “technical thing”) construct and shape the way scientists see and organize what he refers to as the “epistemic thing” (about which knowledge is to be produced). Rheinberger argues that both the epistemic thing and the technical thing change in relation to one another. In this view, instruments are more than just simple vehicles for testing a theory, as is often supposed in philosophy of science, but have a productive effect on the epistemic object. Here I propose understanding the term “technical thing” in a broad way as suggested by Mitchell Ash (2007: 14–15) who adapts Rheinberger’s approach for the context of the history of psychology by introducing the term “instrument.” In his conception, the psychological instrument might be an “[a]pparatus as well as routinized methods of observation and data interpretation” (ibid.). Thus, the diary itself can be considered an instrument used by psychologists in order to make the adolescent’s inner life legible. But to do so, they had to know how to use it. I argue that “interpretation technique” would be a suitable term to describe the way psychologists used diaries as psychological instruments. Talking about an interpretation technique presupposes three things: psychologists had to conceptualize which parts of the diary were to be interpreted (isolation of relevant facts), they had to establish an overarching question as the frame of reference for the interpretation (depending on what they sought to know by interpreting a diary), and they had to create principles of interpretation (implying they had to know something about the connection between what is to be interpreted and its significance for the intended epistemological interest). None of the three psychologists explicitly developed such an interpretation technique, but it can nonetheless be deduced from their use of diaries in their selected monographs.

This methodological approach is distinct from that of most authors who write about Bühler, Stern, and Bernfeld. The three are often discussed in the context of institutional or biographical histories, but historians rarely investigate the scientific methods and practices their actors applied.^4^ The collection of self-testimonies has already been the object of scholarly attention; however, the epistemic dimension of diary research has hardly been taken into account.^5^ Additionally, one article (Stach 1994) analyzes how representatives of psychology and psychoanalysis used the object of the diary in youth psychology in order to debate who had the interpretative upper hand in the nascent field of youth psychology.

By adopting an approach informed by historical epistemology, I contribute to a history of psychological research practices and observation techniques as suggested by Mitchell Ash and Thomas Sturm (2007). Furthermore, my article can be contextualized within the history of scientization of youth in the twentieth century which in more general aspects has already been investigated (see for example von Bühler 1990; Dudek 1990). I understand the three psychologists as knowledge producers of a certain scientific idea of (normal) adolescence. Such a perspective must recognize its own limitations, as science was only one of many areas which produced knowledge about this subject. Works on the self-understanding of youth and on youth movements in the 1920s have shown that young people themselves have played a decisive role in shaping the perception of youth as a phase of life (see for example Gillis 1974; Dudek 2002).

## Three Explanatory Models for the Transition Between Childhood and Adolescence

Until the turn of the twentieth century, the child (rather than the adolescent) had been a focus of psychological interest. Parents and psychologists observed children’s behavior and development and documented it in so-called *Elterntagebücher* (parenting diaries), following a practice established in the eighteenth century.^6^ In contrast to this practice centering around the child, research into adolescent behavior had two basic differentiating features: Psychologists could not observe subjects inconspicuously from the outside, and they also perceived adolescents as being different from children. Psychologists and physiologists used the criterion of age in order to distinguish the adolescent subject from the child: From the age of 13 (for girls) or 14 (for boys), one was considered an adolescent.

Besides age, physical appearance could be used to distinguish between these different periods of life: For Bühler, the passage into adolescent life was concomitant with physical sexual maturity and was “completed as soon as the sexual apparatus is ready for use” (Bühler 1923: 9). She called the specific transition from childhood to adolescence the phase of “puberty” or—analogously—“maturation,” with reference to biological concepts, while noting that puberty represented one stage in the whole process of human development (ibid.: 16). “Maturation” for her clearly had a biological function: it was the expression of a predetermined physical need for reproduction. This shows that Bühler essentially conceived of development as something that was induced biologically and thus could be described by natural laws. However, while physical aspects remained the primary determinant for the transformation between stages of development, the physical transformation also brought about “a series of special *mental* phenomena that were related to the biological maturation in terms of meaning and purpose” (ibid.: 10, emphasis in the original). Bühler called this process “mental puberty” (*seelische Pubertät,* ibid.) and based this further distinction between child and adolescent on a changing subject-world relationship. The most important psychological expression of the sexual need for reproduction for her was the adolescent’s “need for supplementation” (*Ergänzungsbedürftigkeit*, ibid.): in this way, she translated the biological need into a corresponding mental one so that the “Me should be opened up for the encounter with a You” (ibid.: 11) during the period of maturation. Before this process of opening up to another person and affirming the outside world could take place, the young person had to pass through an opposing state, a process of isolation and inward retreat. This state was the precondition of another change in the inner world of adolescents: the formation of a “self-awareness” (*Ichbewußtsein*, ibid.: 40) which Bühler saw as the most important psychological function of puberty. According to her, while children live from one day to the next without consciously recording their impressions or reflecting on experiences, adolescents develop the desire and ability for historicization and self-reflection. Due to changes on the physical level, they start to experience themselves consciously as a unit that stands out sharply from the outer world: “I” (ibid.: 40–41). Bühler called this process the “discovery of the self” (*Entdeckung des Ich*, ibid.: 49) which she understood as the idea that the young person started to observe their own thoughts and to reflect on their inner emotional world. This process of self-exploration took place through the act of writing a diary.

In delineating her concept of the “discovery of the self” during the transition period from childhood to adolescence, Bühler followed William Stern (see ibid.: 40–41). The latter had used the term to refer to the supposed ability of adolescents to analyze their own thoughts and feelings (see Stern 1922). But on closer inspection, it is worth noting that she only partially integrated aspects of Stern’s approach into her biological perspective. Even though for Stern “self-awareness” (see ibid.) also signified the threshold between childhood and adolescence, he did not refer to biology in order to explain the phenomenon. For him, “puberty” or the “period of maturation” (*Reifezeit*) was a phase of adaptation to and playful appropriation of the social environment. Within this concept he understood puberty as the internalization (*Introzeption*, ibid.: 8) of external cultural values. The transition between childhood and adolescence took place primarily on the mental, not the physical level: through individual mental conflicts, self-reflection was being set in motion (Stern 1925a: 22). According to Stern’s theory, this mental state encouraged the tendency to translate the preoccupation with oneself into linguistic expressions, such as those found in a diary. Since it was not, as with Bühler, the physical that acted as motor for changes on the mental level, but rather the non-physical processes which primarily drove the dynamics, Stern ascribed more importance to the individual and social context of a person in order to explain the transition that he also referred to as “development” (ibid.: 3). Like Bühler, he regarded his work on adolescence as a contribution to developmental psychology with the aim of identifying regularities and definable phases in human life. He strove to isolate typical psychological characteristics of childhood and adolescence and separate them from individual ones. However, he differed from Bühler in his theoretical approach, which he himself described as “personalistic”: “The individual expressions of the soul should not be described phenomenologically for their own sake or segmented into their simple elements as in elementary psychology, but rather captured in their personal significance” (ibid.). The phrase “personal significance” highlights a position that he had already developed between 1900 and 1902 in connection with his studies of child psychology (see Heinemann 2016: 149) and in which the concept of the subject as “‘*unitas multiplex*’, a multi-unit” was central (Stern 1925c: 28, emphasis in the original). In this approach, the human subject was understood as an indivisible whole consisting of complex psychic and physical processes (Stern 1927a: 166). Stern described his so-called critical personalism as an approach that “seeks to understand everything psychic in its significance for the unity of the personal life and for the determination of personal development” (Stern 1925c: 28). In connection with Stern’s youth psychology, this approach meant that a single subfield, such as the maturation of sexual functions and their physical and psychic consequences, could not be elevated “to the place of origin and explanation of that total change [between childhood and youth]” (ibid.), as it appeared to Bühler.

The distinction between childhood and adolescence was not as clearly delineated in Bernfeld’s theory, since he pursued different research interests and, thus, did not focus on developing a comprehensive or explicitly formulated theory of development. In his concept, drives served as the motor of behavior—in this sense he borrowed his theory from Freud. But these drives for him never could sufficiently explain in which way individuals interacted with their environment. Bernfeld’s perspective differed from Bühler’s, in the sense that he was not interested in (biological) processes inherent to the individual, but wanted to investigate the interplay between inner drives and the ways they could be articulated which for him were conditioned through society or culture. He wrote in a marginal note that his thinking showed important points of contact between the psychoanalytic and the personalistic view of Stern (Bernfeld 1931: 39, FN 1). Indeed, much like Stern, Bernfeld’s theory probed how external cultural values are internalized individually, as well as vice versa: how individuals become part of the specific culture they live in. With reference to Freud, Bernfeld assumed that each individual participates in an “inner synthesis” (ibid.: 36) of the self. This is achieved, for example, by cultivating a sense of continuity with one’s own past and historicizing it, by integrating one’s own goals and desires, and by setting boundaries to other people. The “integration and contouring of one’s own personality” (ibid.) takes place in a mental process of reflecting and fantasizing. During this process, the subject creates an imaginary ego—for example by writing a diary or an autobiography—shaped by desires, fantasies, and ideals and has to constantly compare this self-conception with reality in order to bring the two into accordance (ibid.: 40). However, Bernfeld did not necessarily see these processes taking place during puberty (ibid.: 39), although this period of life more often stimulates work on the inner self, as young people find themselves challenged by many questions concerning an uncertain (economic and professional) future, or their place in society and family (ibid.: 38).

To sum it up: Bühler, Stern, and Bernfeld attempted to explain the changes in the behavior of young people during the period of transition between childhood and adolescence. They agree that reflection on one’s own inner life constituted an essential practice accompanying the process of becoming an adolescent. This inwardness was described in different terms and the motors of inner change are explained differently. Stern and Bühler understood inwardness to mean the feelings, thoughts, imaginations, memories and wishes of the adolescent subjects that in part motivate their actions. They summarized these aspects in their writings under the term “soul” (Stern) or “soul life” (Bühler). Also, both posited the idea of the “discovery of the self” as a central criterion in order to distinguish between childhood and adolescence. In general, it can be said that developmental psychology provided an important frame of reference for both approaches: they focused on making visible inner-subjective processes of change that could be described as standardized processes. Despite such commonalities and the fact that Bühler and Stern shared similar terminology, they differ in the way they conceptualized the forces stimulating changes in internal life. This became very clear by the fact that for Bühler the “discovery of the self” was induced biologically, whereas for Stern it was stimulated by the social environment. In this respect their approaches are quite different: whereas in Stern’s personalistic view the motor for inner change comes from the adolescent’s social context, in Bühler’s approach change is induced by the physical needs of reproduction that also drive changes on the mental level. Thus, it can be said that Stern’s way of looking at adolescent subjects was actually closer to Bernfeld’s thinking. For both Stern and Bernfeld, the interplay between outer world (society and culture) and inner adaption was important. They were more interested in how individuals internalize cultural values and thus become integrated into society and adopt its norms as opposed to viewing the individual as a biological entity, as Bühler did. Stern and Bernfeld also agreed that adolescents had no direct access to their inner world and, accordingly, that their conscious perception of their own thoughts and feelings was not always credible. In Stern’s conception, a secret world was hidden behind the consciously perceived expressions of the adolescent’s “soul,” which he called the “true self” or “essence” (*Wesen*, Stern 1927b: 9). Bernfeld uses the term “virtual self” (*virtuelle[s] Selbst*, Bernfeld 1931: 40) in order to describe the inner life and thus immediately makes clear that he considered the perceived inwardness to be a projection. In his view, the adolescent’s perceived inner life was made of experiences, feelings, ideals and wishes which were not always congruent with reality and influenced the self-perception of the young subject.

I have already shown that the practice of writing played an important role in connection with the theoretical idea of self-exploration. The next section explores the connection between diary writing and the conception of the young subject in the transition from childhood to adolescence. It focuses on the different understandings of the status of the diary as testimony for the adolescent’s inner life between the three authors. In one view, it could serve as an opportunity to follow a complete life cycle between two book covers, while for others, namely for Stern and Bernfeld, diary descriptions could not be trusted uncritically; they only gave hints that required interpretation in order to access the adolescent’s hidden inner life.

## Reading a Book of Life or Doing Detective’s Work? Why Interpreting a Diary Does Not Always Mean the Same Thing

According to Bühring (2007: 77–78), Charlotte Bühler’s monograph *Das Seelenleben des Jugendlichen,* first published in 1922 and then reissued four more times until 1929, was one of the most successful books in the field of youth psychology in the 1920s. Within a few years, Bühler had become a renowned psychologist in the field of youth psychology. While at the Vienna Psychological Institute, she had collected 130 diaries^7^ by 1938.^8^ In the preface to the second edition of the* Seelenleben*, she articulated the promise of diaries as sources as follows:

For the time being, I can think of no better source than the diary […]. A diary that has accompanied a young person in lonely struggles, in his youthful distress and longing, and his youthful happiness over the years, leaves a deep impression of the value of human life and of the importance of the developmental years in particular. (1923: VIII)

This passage already indicates what Bühler expected from the diary: to enable her to follow a life over years in almost real time and directly, “not inhibited by any barrier of convention or custom” (ibid.: 35). It was precisely the staging of temporality characteristic of the diary genre (see Dusini 2005: 9–10) that Bühler saw as enabling her to re-experience the inner life of the youth, as it suggested immediacy and fostered the illusion that the distance between real life and life on paper had been collapsed. For Bühler, the promise of seeing the totality of a life unfold made the diary distinct from the experimental method, which in her eyes only accessed parts of the young subject “from the outside” (Bühler 1923: 7). Bühler therefore called the diary “a developmental book” (ibid.) that made deep processes of the inner life visible:

In addition to the directly presented details it [the diary] shows us *developmental facts* and a *direction of development*. However, we cannot read them as easily as the facts themselves. It is also not possible to follow them by measurement like the development of body length or body weight. Here, qualitative individual facts must be juxtaposed and their meaning and developmental context must be understood by interpretation. (ibid., emphasis added)

Bühler treated the diary as a direct testimony, which not only provided access to the inner state of a specific individual, but also to a more general conception of development. She sought out evidence of development itself in the diary. This presupposed knowing which contents of the diary were meaningful for the adolescent’s development and thus worthy of interpretation. In her case, she brought an external frame of reference to the material: it consisted in a “theoretical preparation” (ibid.), namely in her law of puberty outlined above. Bühler saw development as preconditioned by biology and drew on the analogy of physical and mental puberty and the need for reproduction and supplementation. As mentioned before, this process consisted of two phases: first of isolation from the outside world and then of re-opening towards the outside and turning to a partner. The isolated state of mind in the first phase provided the necessary condition for writing a diary. Since Bühler saw the conditions for writing a diary as identical to one of the main characteristics of puberty (Bühler 1925: IX), she could argue that writing a diary fulfilled a function in the developmental process by helping adolescents satisfy their compulsion toward self-preoccupation and historicization resulting from the “discovery of the self”. Bühler thus concluded that “diary writing originates from a necessary and specific experience of the age of maturation” (ibid.: X). For this reason, she considered it a phenomenon following the natural course of development as determined by biological and psychological determinants, and at the same time also as an expression and vehicle of development. The close relationship between diary writing as a phenomenon and Bühler’s model of development was further shown by the fact that she equated the end of the writing practice with the fulfilment of the mental need for supplementation: as soon as individuals reached the stage of development and found a partner, most concluded their diaries (Bühler 1923: 66–67). As Bühler had already identified the basic structure of development, the diaries she cited in her monograph could only show “the manifold ways in which the law [of development] appears” (ibid.: 7). Her approach can therefore be described as casuistry, as knowledge production that reflected a more general law through the examination of individual cases. For Bühler, the diary served primarily as an example, while it also allowed her a greater degree of differentiation between possible manifestations of the adolescent course of development in order to map out all of these varieties.

In this respect, her approach resembled that of Stern, who also wanted to illustrate the general characteristics of puberty. However, Stern concerned himself not with developments on a biological level that acted as preconditions for changes on the mental one, but with different relations between the adolescent subject and the outer social world (family, friends, intellectual interests) as consequences of the newly acquired self-awareness. Unlike Bühler (1923), who drew on fourteen different diaries for her study, Stern centered his analysis around the diaries of one single boy. Stern challenged Bühler’s conceptualization of the diary as a “book of life.” He criticized her approach by saying that diaries were not “adequate soul portrait of the authors” (Stern 1925a: 2), but materials which only gained significance through interpretation:

Here, too, one must interpret and reinterpret; one must often read between and behind the lines—and above all, one must not regard what is not said in the diary as missing or emotionally irrelevant. For there is also a shame before oneself, which sometimes makes it impossible to write down essential things. (ibid.)

According to Stern, what was written in the diary was not “an immediate proclamation of mental realities” (Stern 1927b: 9). Instead, he stated that the diary could not be a reflection of an entire life totality, since he considered writing to be a selective process (1925a: 89). Additionally, his view of the diary as an object to be interpreted was also based in his conception of the adolescent subject.

Stern generally regarded the young person as more opaque than the child or the adult. He identified a lack of credibility as a typical characteristic of the period of puberty during which “the direct view of what man does and experiences, towards what is actually essentially meant by it, is most hindered” (Stern 1927b: 8). In principle, he posited that the inner nature of the young subject was not directly accessible to them. In this sense, the experienced “intellectual and emotional contents […] are only very partially adequate reflections of his [the adolescent’s] being” (ibid.: 9). The linguistic utterances in the diary have to be interpreted for Stern, because in his view, the personal structure is merely represented as a projection “in physical or psychic symptoms” and thus is itself always “veiled, repressed, symbolized” (ibid.: 10). As a result, the actual meaning of a linguistic utterance or an action remained obscured inside the individual and could not be directly experienced either by the young subjects themselves or by the outside observer. Consequently, it was also possible for Stern to interpret what had *not* appeared in writing; even the blank space could be given meaning. The fact that the diary’s insights with regard to development had to be interpreted did not imply, however, that Stern’s understanding of the connection between diary writing and adolescence completely diverged from Bühler’s: he too was of the opinion that the examination of the self during puberty often led the adolescent to keep a diary in order to “give an account of oneself and to speak out—also to mirror oneself in oneself” (Stern 1925a: 1). His attribution of authenticity to the diary format also resembled Bühler’s, and he similarly assumed that diary keeping was a spontaneous activity that emanated only from inner impulses and was not guided by external influences. However, Stern held that diary writing was not especially central to the developmental process, as only a specific type of adolescent chose to keep a diary in order to provide mental relief and to portray themselves. The individual diarist “A.” and his transition from childhood to adolescence formed the focus of his examination in his 1925 monograph. Stern aimed at identifying the break between these two phases of life in the diary by showing how, at multiple levels—not only in the content—self-reflection was set in motion. For example, he interpreted the change from a regular notebook to the medium of the “agenda,” which regulates the writing frame clearly according to the structure of the day, as a sign of a new mental attitude in the maturing subject (ibid.: 10). The change of medium identified here corresponded with observations of the author’s handwriting: by inserting images of the diarist’s handwriting into his text, he tried to make A.’s development visible in the material itself by detecting a difference between the child’s and the adolescent’s handwriting (see ibid.: 11–12). Thus, “an accent of experience could become visible in writing” (ibid.: 12), which exceeded the semantic level of meaning. Since Stern, unlike Bühler, did not take the writing at face value, he had to find a way to decipher the meaning behind it. To this end, Stern inserted quotes from the diarist, who was now in his fifties, into the text, in addition to his own psychological commentary. The diarist explained and analyzed the notes made by his younger self. The adult perspective takes on the function of a corrective to adolescent memory by explaining some events in greater detail, pointing out gaps in what had been written down, engaging in temporal anticipations (see for example ibid.: 37, 95), and comparing different memories (see for example ibid.: 5).

Despite the fact that Stern—unlike Bühler—problematized the credibility of the diary’s contents, he also retained some idea of authenticity with regard to the phenomenon: First, he was convinced that something true could be detected from the diary’s content, provided that the thoughts of the adolescent were compared with those of the self-aware and trustworthy adult. Second, he followed Bühler’s assumption that diary writing was a spontaneous activity that originated in natural inner impulses of puberty. While for Stern the contents of the diary required interpretation, the activity of diary writing remained deeply private and internally motivated, as there was “hardly any activity that would be so little forced and influenced by circumstances outside of the self as keeping a diary” (ibid.: 1).

Here, Bernfeld went one step further in his problematization and broadened the perspective by not only casting doubt on the credibility of the diary content as Stern did, but also critically questioning the choice of the diary as a medium, which Stern and Bühler had understood as “natural.” For him, the question of why adolescents write diaries could not be explained by individuals’ interior lives. Adolescents’ choice to keep a diary was thus dependent on their milieu and contemporary trends and could not be considered without inviting larger considerations related to social class and power relations. This leads us to an important aspect of Bernfeld’s diaristic theory: He perceived psychology as always intertwined with political questions that were informed by his engagement with the Youth Culture Movement (*Jugendkulturbewegung*). Bernfeld’s critical, Freudo-Marxist view on society informed his opinion that instinctual life represented a “sediment of historical events” and that “‘reality’ for humans is almost entirely given by society” whereas “‘nature’ plays a minor role here” (Bernfeld 2012 [1929]: 255–256).

He further developed this idea in his 1931 monograph, which dealt with the idea of tradition. His interest in knowledge accordingly articulated itself in a kind of cultural-historical approach. Bernfeld analyzed how varying social conditions gave rise to different cultural forms of writing and how these different forms spread socially (Bernfeld 1931: 108–124). What Bernfeld then focused his investigation on was a “psychology of the diary” (ibid.: 3–4), which offered an answer to the following questions:

What causes today’s young person, today’s child, to adopt the literary custom of the “diary” for himself? How far does he adapt his diary entries to a norm? What causes this adoption of this form? What does the form and its adoption mean to him psychologically? (ibid.: 3)

The fact that Bernfeld’s interest was directed towards the adoption of culturally determined forms in a specific epoch reveals two points: Both the development of various literary forms and the connection between the so-called adoption of form and the satisfaction of needs in his eyes were historically and socially variable. For Bernfeld, the choice to keep a diary was not a necessary consequence of development as it was for Bühler and, to some degree, Stern. This also influenced his perception of the diary as testimony: It no longer merely provided traces of a subjective inner life, but also traces of the social world the subject inhabited.

In Bernfeld’s view, writing a diary was only one of many ways to satisfy certain needs. Sometimes these needs even coincided with those that had been identified by Stern. For example, Bernfeld agreed with Stern’s thesis that in puberty “a greatly increased affinity for autobiography” (ibid.: 36) is found, as this practice of self-representation could serve as a suitable means to gain self-awareness and represented one possible path to “inner synthesis.” However, Bernfeld did not derive a general law from this observation. Individual needs that were satisfied through writing a diary could also surface before the time of writing, and in this sense the beginning of the diary represented “only a change in the form of the previous writings, in the previous ways of coping with those needs” (ibid.: 75). All this mirrored his theoretical stance that diary writing was embedded in a social setting; it presented one possible medium of drive satisfaction that was chosen because familiarity with this form had been culturally transferred. In order to illustrate this point, Bernfeld historicized the form of the diary and asked about its historical predecessors, which he described as “basic forms” (ibid.: 74). After identifying them, he theorized about the various psychological functions they could have for the writers. Such basic forms were for example 1) the collection of relics, 2) the autobiography, 3) the letter, and 4) the register of sins and virtues. In all of these forms he identified certain aspects of form which were also found in the diary in different combinations: 1) the form of collecting “souvenirs, fetishes, relics” (ibid.: 17) with symbolic value, 2) the form of retrospectively summarizing and depicting a holistic experience with a constitutive artistic moment (ibid.: 31–32), 3) the form of the dialogical orientation of a written self-portrayal to another person (ibid.: 4, 46), 4) and the form of moral balancing and taking inventory (ibid.: 55). Bernfeld worked out different functions of each form and the associated satisfaction of needs. To sum it up, Bernfeld regarded diary writing as a reflection of the adaption of norms, the attainment of absolution through the confession of misdeeds in the diary (see for example the comment on “masturbation registers,” ibid.: 102), a narcissistic and a libidinous identification (see for example the case Hedwig, ibid.: 28–29), and the fulfilment of repressed desires (see for example the case Irmgard Winter, ibid.: 50–52).

It is important to note here that Bernfeld did not systematically break down these functions in his monograph, but instead dealt with them by analyzing individual cases. Regarding the cases, Bernfeld asked why the subject began to keep a diary and distinguished between motives of voluntary and forced adaption of the practice. In doing so, he tied his research on instinctual life back to certain socio-cultural conditions. If a diarist voluntarily took up the form, the choice originated from a cultural “knowledge of diaries” (ibid.: 76), which had been appropriated—for example by reading literary models—and was then affirmatively imitated. For example, he interpreted the diary writing of the young boy Ortmann (ibid.: 74–82) as the expression of his wish to be a “great, important man.” According to Bernfeld, this was only possible because Ortmann was impressed by Goethe (who he often mentioned in his diary) and his writings, and had associated being an important man with developing a writing practice. Thus, diary writing became a means of identification with his idol and satisfied Ortmann’s wish to emulate.

Yet diary keeping could also be forced upon young people. In this case, parents or teachers exerted pressure to write in a diary, with the aim of cultivating a “good conscience” (ibid.: 126) by adapting to the norm. This was the case for a diarist called Maria (ibid.: 91–96) who was not very consistent in her efforts: she started writing her diary four times without continuing it. Bernfeld interpreted this pattern as an “urge to be obedient to her mother and her mother’s stimulation” (ibid.: 95). Maria received the diary as a present from her mother, with whom she had a complicated relationship, and in Bernfeld’s view, the girl returned to the diary when she felt the need to be attached to her mother again and to cope with her ambivalent feelings towards her. Bernfeld’s interpretations of his cases make clear reference to Freudian psychoanalysis (see for example ibid.: 88, 95) and can be further contextualized within this framework. Bernfeld perceived diaries as “representations distorted by conscious and unconscious tendencies, just as dreams, fantasies, poems of young people” (Bernfeld 1927: 39). In his opinion, they provided the psychoanalytically trained reader with two insights:

1) the knowledge of the manifest feelings (i.e., manifoldly distorted by tendencies), desires, and experiences of puberty; 2) that they are sources for the interpretation of these tendencies and the psychic material distorted by them. (ibid.)

Bernfeld used the term “manifest” to describe the content that appeared in diary entries and could be interpreted at the semantic level. Diary interpretation required interpreting the manifest content with reference to the individual and personal contexts he detailed in his monograph. With his use of the term “manifest,” Bernfeld implicitly referred to the idea of the “manifest dream content” (*manifeste[r] Trauminhalt*) formulated in 1900 in Sigmund Freud’s* Traumdeutung *(Freud 1961 [1900]: 140). Freud used this term to refer to the dream that is accessible in memory, through which a way to the “latent dream content” (*latente[r] Trauminhalt*, ibid.) behind the dream could be found by means of analysis work. Freud’s idea of the interpretation of dreams, which can only take place with “the help of the associations that the dreamer himself provides to the elements of the manifest content” (Freud 1955 [1938]: 92), can thus be read in parallel with Bernfeld’s demand that the collection and publication of accompanying material are indispensable for the interpretation of the diary.

## What Information Do Psychologists Need to Interpret Diaries? The Controversy Surrounding Publishing Practices and the Use of Diary Sources in the Monographs

In the previous section, I showed that Stern and Bernfeld, who did not regard the diary as a direct testimony of adolescents’ inwardness, resorted to various auxiliary constructions in order to arrive at an interpretation of the diaries. I will now examine how these actors discussed the question of how to use diaries for psychological research and show how a controversy on publishing practices revealed different views on the topic. This debate was touched off by Bernfeld’s critical stance on Bühler’s work.

According to Bernfeld, Bühler’s interpretation technique did not bring about any theoretical progress, but only served to recapitulate what the diarists had already expressed themselves (Bernfeld 2010a [1928]: 272). His polemical reproach referred to her *Seelenleben,* where she grounded her assertions based on the frequency of similar evidence. To support her classification of characteristic features of puberty derived from theory, she grouped examples from different diary entries together, either citing them in detail (see for example Bühler 1923: 76) or simply indicating the signatures of the corresponding diaries (see for example ibid.: 28). Bernfeld charged that Bühler did not further theorize and contextualize diaries and diary writing, but instead saw her knowledge production already completed in and through the diary and its content. With reference to Ash (2007), one could say that for Bühler, the instrument of her research that shaped the view on her epistemic object—in this case, the adolescent’s development—was primarily her theoretical structure (which was based on biological models) and not the diary. The latter neither influenced her production of knowledge nor did it change the idea of her epistemic object. Bernfeld further criticized her for failing to add “*psychographic information* about the author” (1928: 174, emphasis in the original). Here he referred to the question of how diaries should be contextualized in order to make them useful for psychological research. Two different approaches to the diary are expressed in the two publications: For Bühler, contextualizing information about the diarist was only of limited interest. She perceived of the individual diarist as a type that only illustrated a more general law (I described this approach earlier as casuistry). Therefore, beginning with the second edition of *Seelenleben*, she added an overview table of the source corpus used before the text, in which she listed signatures given to the individual diaries, noted the gender of the diarists and named the profession of their fathers. Bühler’s disinterest in the diarists as individuals within a specific historical context is further underlined by the fact that instead of indicating the writer’s year of birth, the only temporal information given in the table was the “period of diary keeping,” expressed in years of life. A category titled “course of development” summarized the diarist’s educational career, while the writer’s home and background were not indicated at all. By presenting the information in such a way, Bühler inscribed the idea of ahistorical development into the table. By erasing the writer’s context, she succeeded in transferring the diaries into a new reference frame of biological development. This specific use of the diaries helped her not only to unify and standardize her corpus, but also to make the individual sources comparable and thus subject to her development model. All of the aspects of Bühler’s use of diaries recalled practices in the natural sciences, marking a stark contrast from Bernfeld’s—and in parts also from Stern’s—understanding of the diaries’ use.

Bernfeld presented his interpretation of several diaries by providing extensive introductions to the writers in his 1931 monograph. As I have shown, in his view a young person’s social context, the reasons why someone wrote a diary, and whether they knew of other diary examples provided vital information for interpretation. He also sought to publish diaries as source materials in full length, as every word could be important for interpretation (see Bernfeld 1927: 38). Additionally, he demanded that even letters and other written material should be taken into account in order to get a full impression of the diarist’s personality and to then evaluate the meaning of their diary in relation to this overall picture (Bernfeld 1928: 174–175). As he was aware of the editorial burden of such a process, he suggested something rather more pragmatic: psychologists should use already existing sources published for the purposes of historical or literary studies, such as diaries and biographies of poets and scientists (ibid.: 175). In several publications (1928; 1931) he then listed publications of diaries that in his eyes could be used for psychological purposes and even published two diaries himself in the appendix of his 1931 monograph. His demand for contextualizing material, however, as well as his problematization of the diary, diary writing and its history, which was put into position against Bühler’s use of diaries as simple and “objective” sources, can be read as expression of a different research method. Bernfeld aimed at suggesting a path between cultural-historical and psychoanalytical perspectives and tried to integrate sociological and psychological questions. However, according to Dudek (2012: 155), this view of youth studies as a multidisciplinary project was not well received in contemporary academic circles, as it did not fit into the conventional structure of the research landscape.

Stern also advocated for a biographical contextualization of the diarists: precisely because what was written was not self-explanatory and always went beyond the semantic content, he considered it insufficient to publish diaries without interpretation and biographical contextualization (Stern 1925a: 2). For this reason, he dedicated a whole chapter of his 1925 monograph to introducing the diarist “A.” and his biography (ibid.: 4–9). Here, he described the roles of different family members and analyzed constellations of people and their potential for conflict (which were important as he assumed that “mental conflicts” set self-reflection in motion (see ibid.: 24)). Although Stern wanted to illustrate general characteristics of puberty in A., his interpretation technique allowed room for A.’s individuality and his specific responses to various phenomena, which he explained within his theoretical frame of critical personalism. For example, Stern describes A.’s “mental behavior during particularly exciting events” (ibid.: 29–34). Here, A.’s experiences with death and illness of relatives are placed in the foreground. However, in most of the cases Stern does not consider A.’s reactions to be “typical” of adolescents, as Bühler did. He also included diary quotations in his monograph in a different way than her. This had to do with the fact that he carried out his analysis based on a single diary and did not work with the frequency of evidence as she did. Stern was less interested in theorizing a law of development that prescribed a specific series of developmental events in a temporal structure. Thus, we see Stern’s methodological positioning of youth psychology between the natural sciences and the humanities which Heinemann (2016: 296) has also pointed out. With reference to Bühler, Stern criticized that it was not enough to anchor youth psychology in the natural sciences, but that the methods had to be supplemented with tools of humanistic inquiry, such as interpretation. Although Stern was interested in regularities of development, he understood development as a process that involved deeply interlinked socio-individual and biological factors. In principle, the focus was on the person and their social context as a frame of reference. This is also evident when analyzing the relationship between quoted diary entries and the author’s psychological comment, as a large part of Stern’s monograph consists of detailed and extensive quotations from the diary entries, while Stern’s interpretive commentary often takes a back seat.

Stern’s publication was positively received by Bernfeld (2010b [1928]: 273–274), as he saw his claim of “psychographic” contextualization realized here. He only criticized two aspects: First, he lamented that Stern only had published excerpts of the diary instead of publishing it in full length. Second, he noted that as a result, the diary material was entangled with Stern’s own interpretation and was thus not useful to other researchers. This was probably one of the few points on which Bühler and Bernfeld had ever agreed. Bühler (1927: VI) also underlined that she could not use the diary material published by Stern for her purposes and pleaded for more diaries to be published without interpretation as source material for other researchers. These demands for an “objective” publication of diaries as source material for other researchers shows that by the 1920s and 1930s, the diary had become an established object for psychologists. However, it also underlines that the interpretation of diaries was a controversial subject in which no one wanted to give up epistemic legitimacy: diaries were to be published unedited so that everyone could apply their own interpretations.

## From Diaries to Interviews: the Rise and Fall of a Psychological Instrument

In this article, I investigated how the psychologists Charlotte Bühler, William Stern, and Siegfried Bernfeld interpreted and theorized young people’s diaries to produce knowledge on the inner lives of adolescents in the 1920s and 1930s. I started with the question of how the psychological use and interpretation of diaries could be contextualized within the still undefined field of youth psychology. With reference to the cultural history of diary writing, I explored the reasons that made diary writing an attractive instrument for psychological research. A closer look at the three actors shows that they all used diaries for their research, but treated them in different ways with respect to their theories on adolescence, their interpretation of diaries and their styles of publication. The comparative examination of the underlying concepts of adolescence has already revealed the different theoretical focus of the actors studied. Against this background, I sharpened the various interpretative frames of the phenomenon of diary writing: I showed that Bühler did not really interpret the diaries, but rather used them as illustrations of a law of adolescent development she had borrowed from biology. Stern, like Bühler, understood his approach as casuistry, but it differed from hers in two crucial points: first, he explicitly did not formulate a general law of development that unfolded in a temporal structure and, second, he interpreted the diary material with greater reference to the individual and personal circumstances of the diarist. Bernfeld’s interpretation technique was an implicit one; in his “cases” he offered examples of how to analyze diaries, but did not systematically depict such a method. While he asserted that the question of how to interpret diaries in psychology remained undecided (1928: 174), Bühler positioned herself confidently. She just claimed to have been the first to analyze diaries as a “methodological principle” (Bühler 1925: V) for youth psychology. The fact that other psychologists trusted her self-narrative had several reasons: First, her social position in science—she was not only well connected in Vienna, but also received funding for her research^9^—ensured her visibility and scientific authority, while Bernfeld remained an outsider throughout his life. Stern, on the other hand, was a well-established academic psychologist, however his contributions to youth psychology was rarely noted. Second, Bühler’s theoretical reference to biological concepts was more compatible with the mainstream of psychological research of the time. This contributed to the positive reception of her work.^10^ Third, she was more visible than the others through her publishing activity: Bühler had systematically published diaries of writers from her Vienna collection in unabridged versions as source material for other scholars since 1922. All these aspects enabled her to successfully promote the idea that diaries were important material for psychological knowledge production on adolescence. The term “diary method” that she coined (see for example Bühler 1927: VII) could thus become attractive for other actors who wanted to secure their position in youth psychology.

Due to the popularity of the “diary method,” the diary became an object with reference to which psychologists thematized academic and epistemic legitimacy. For the historical analysis of psychological techniques, this means that these discussions about methods or interpretative techniques point to more general controversies. The diary enabled the actors to discuss who could interpret youthful inwardness with authority (psychoanalysis or psychology). Furthermore, psychologists addressed the self-conception of youth psychology as a discipline: for Bühler, psychology’s methodological orientation lie in the natural sciences, while Stern and Bernfeld sought to integrate perspectives and methods of the natural and social sciences with those from the humanities. Finally, the debate provided the opportunity to discuss who was regarded as a legitimate authority for scientific knowledge about adolescence and whom it served: while for Bernfeld it was an emancipatory project of the youth themselves, for Bühler it presented a possibility to set a research agenda and establish her newly founded institute.

The idea that the diary was an appropriate source to analyze youthful inwardness did not automatically emerge from the research process. In order to understand how the diary entered psychological research, one must take the broader cultural landscape into account. In the 1920s, the diary existed as a culturally preformed object that corresponded to psychological research or maybe even stimulated it. One can even say that Bühler simply took up existing ideas on diary writing and presented them as biological facts. The ideas of self-exploration and self-reflection, which were already strongly bound to the diary in its religious context, were theorized in Bühler’s and Stern’s monographs under a new heading: the “discovery of the self.” The latter was considered a typical theoretical criterion for the differentiation between childhood and adolescence, but at the same time it justified why diaries were ideally suited to understanding the inner life of young people.

Bernfeld also contributed to the idea of making inwardness readable in written expression, but he pointed to the situatedness of this epistemological technique: in 1931 he posited that new technological innovations would develop new forms of expression and new practices and traditions that would group around them. He was convinced that “the telephone and the increasingly widespread oral communication made possible by radio” would “noticeably reduce the importance of writing as a value of tradition and absolution” (144). Indeed, the diary had hardly any significance for youth psychology after 1945 (see Dudek 2012: 154). We can offer an explanation for this loss of significance by looking at Bühler’s preface to the re-edition of her *Seelenleben* in 1967. While she still understood diary reading as a complementary psychological approach, she compared the diary with the interview material the trained clinician recorded during therapy sessions. Bühler now only considered the new form of conversational therapy, “to be able to explore the truthfulness of the expression […] and to get to the actual core of a human being” (Bühler 1991 [1967]: 14–15). She no longer wanted to find the truth about the inner life of young people in written pages and instead sought out the verbal statements in vocal recordings. How this change in the perception of psychological observation techniques took place remains to be examined by future researchers. I have shown that in order to understand this transformation epistemologically, one must investigate the cultural ascriptions to orality and writing, and contextualize them within a world of new technical possibilities, as well as changes in psychological theory and practice.

## Footnotes

1 All translations from German quotations into English are the author’s.

2 See Stern (1922; 1923; 1924; 1925b; 1925c; 1927b).

3 See for example Bernfeld (1931); Boerner (1969); Corbin (1992); Gerhalter & Hämmerle (2015).

4 This article is indebted to the existing work on the three psychologists. These include Gerhard Benetka’s monograph (1995), a comprehensive contribution to the social and theoretical history of the Vienna Psychological Institute in the period 1922–1938; as well as Rebecca Heinemann (2016), who provides us with the first broad account of the scientific activities of William Stern as pioneer of child and youth research. Peter Dudek has published several important works on Siegfried Bernfeld (for example 2002, 2012). A new, annotated edition of Bernfeld’s complete works in 16 volumes has been published by Ulrich Herrmann (since 1991).

5 For example, Li Gerhalter (2021) focuses mainly on the way diaries were treated as objects of collection. She is less interested in the theories and epistemologies underlying diary research.

6 See for example Jaeger (1985); Gebhardt (2015).

7 Recent research has cast doubt on the reliability of the figures given by Bühler. Gerhalter (2021: 158–160) assumes that the inventory of Bühler’s collection could probably have comprised at least 209 records.

8 For further information on the collection, see Benetka (1995: 40); Gerhalter (2021: 243–244). Bühler acquired her material primarily through calls for submissions in the various editions of her monograph *Das Seelenleben des Jugendlichen *(see for example Bühler 1923: VIII).

9 The Psychological Institute was financed partly by the municipality of Vienna and partly by state funds (see Bühring 2007: 60). From 1926 or 1927, Bühler’s work was additionally financed from funds that came from the circle around what would become the Rockefeller Foundation (see Benetka 1995: 61).

10 This can be seen, for example, with regard to Oswald Kroh’s positive review (1927) of her 1922 source edition. The psychologist and pedagogue Kroh also promoted biological concepts in psychology/pedagogy.
